# Electric control of ionic transport in sub-nm nanopores†

**DOI:** 10.1039/d1ra01089a

**Published:** 2021-04-13

**Authors:** Anping Ji, Yunfei Chen

**Affiliations:** School of Mechanical Engineering, Jiangsu Key Laboratory for Design and Manufacture of Micro-Nano Biomedical Instruments, Southeast University Nanjing 211189 China ji-anping@sanxiau.edu.cn; School of Mechanical Engineering, Chongqing Three Gorges University Chongqing 404100 China

## Abstract

The ion transport behavior through sub-nm nanopores (length (*L*) ≈ radius (*R*)) on a film is different from that in nanochannels (*L* ≫ *R*), and even more different from the bulk behavior. The many intriguing phenomena in ionic transport are the key to the design and fabrication of solid-state nanofluidic devices. However, ion transport through sub-nm nanopores is not yet clearly understood. We investigate the ionic transport behavior of sub-nm nanopores from the perspective of conductance *via* molecular dynamics (MD) and experimental methods. Under the action of surface charge, the average ion concentration inside the nanopore is much higher than the bulk value. It is found that 100 mM is the transition point between the surface-charge-governed and the bulk behavior regimes, which is different from the transition point for nanochannels (10 mM). Moreover, by investigating the access, pores, surface charge, electroosmosis and potential leakage conductance, it is found that the conductive properties of the nanopore at low bulk concentration are determined by the surface charge potential leaks into the reservoir. Specifically, there is a huge increase in cation mobility through a cylindrical nanopore, which implies potential applications for the fast charging of supercapacitors and batteries. Sub-nm nanopores also show a strong selectivity toward Na^+^, and a strong repellence toward Cl^−^. These conclusions presented here will be useful not only in understanding the behavior of ion transport, but also in the design of nanofluidic devices.

## Abstract 2

The ion transport behavior through sub-nm nanopores (length (*L*) ≈ radius (*R*)) on a film is different from that in nanochannels (*L* ≫ *R*), and even more different from the bulk behavior.

## Graphical abstract

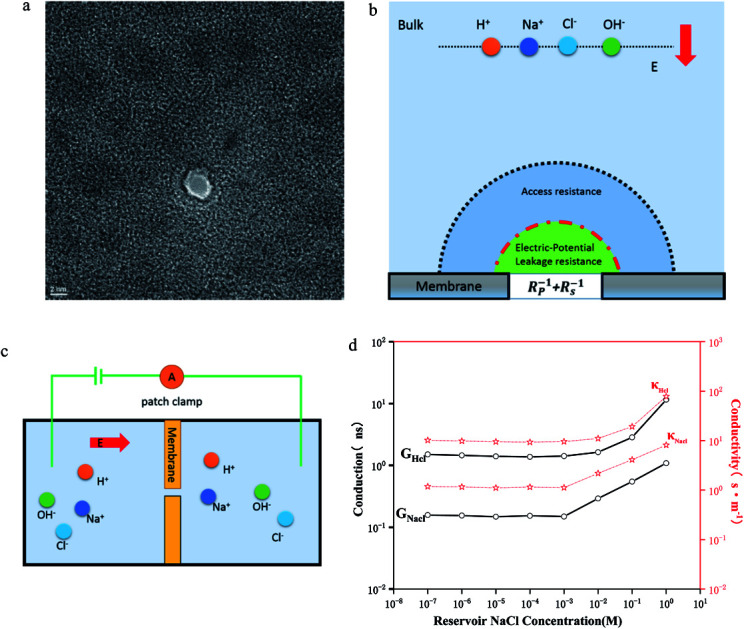

## 1. Introduction

Due to many intriguing phenomena in ionic transport, for example, ion selectivity,^1,2^ the ionic field-effect,^3^ and ionic current rectification,^4^ the design and fabrication of solid-state nanofluidic devices have elicited increasing attention in both the scientific and engineering communities.^5–7^ Electric control of ionic transport is of primary importance for the design of novel nanofluidic devices, such as sensing devices,^8^ water desalination,^9^ and energy conversion.^10^ Many studies have shown that ionic transport in nanoconfinement is mainly controlled by the following factors: (1) geometry, (2) surface charge, (3) chemical composition, (4) wettability, (5) environmental pH, (6) electrolyte concentration gradient, (7) ion mobility, and (8) electric field strength.^11^ Factors 1–4 are determined by the design and fabrication of nanopores, while factors 5–8 can be tuned in the electrolyte solutions. Determining how to exploit these factors in subtle ways is key to the design of nanofluidic devices.

Ion transport is the result of steric interactions (range, 0.1–2 nm), van der Waals forces (range, 0.1–50 nm) and electrostatic forces (range, 1–100 nm).^3,12^ Ion dynamics in a charged nanopore with dimensions comparable to the Debye length (sub-nm nanopore) deviate from the bulk values.^13,14^ Similar phenomena, referred to as charge overspill,^15^ and electroneutrality breakdown,^16^ have been extensively reported. Furthermore, the fluid mechanics in nanoscale conduits have not been fully explored, as they are difficult to measure due to their tiny scale.^17^

It has always been a goal of scientists to manipulate ion transport like that in biological channels (sub-nm), but the scaling behavior^18^ of ion transport is the theoretical basis for this pursuit. Although the continuum theory cannot describe the results perfectly for many experiments, it still has great significance for the modulation of parameters in the design of nanofluidic devices, such as graphene–Al_2_O_3_ nanopore sensors for the detection of DNA.^19^ However, these scaling behaviors^18–24^ have not been evaluated in an in-depth analysis of the nanopore conductance composition. The roles of access conductance, potential leakage conductance, surface potential conductance, nanopore conductance and electroosmotic conductance still need to be further explored. In this paper, we have investigated the ionic transport behavior of sub-nm nanopores from the perspective of conductance using the molecular dynamics (MD) and experimental methods. It is found that the nanopore conductive properties at low bulk concentration are determined by the surface charge potential leaks into the reservoir. At low ionic concentrations, the ion concentration is controlled by the surface charge, and the ion mobility is much higher than in the bulk.

## 2. Methods

**Fig. 1:**
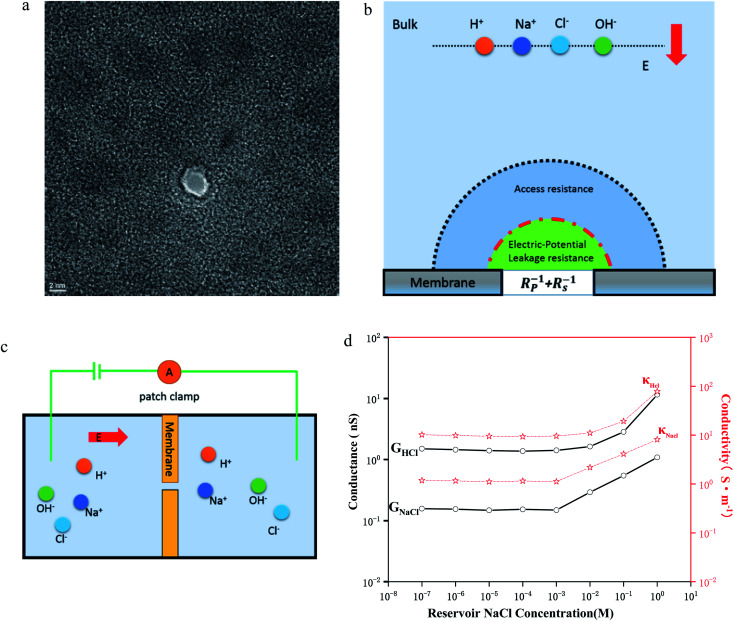
Experimental setup for measuring the ionic currents through sub-nm nanopores. (a) TEM image of a sub-nm nanopore (*D* = 2.2 nm, *L* = 22.0 nm). (b) Schematic illustration of ionic transport through a conical nanopore. The salt solution contains four species, OH−/Cl−/H+/Na+. The ionic transport induces an ionic current through the conical nanopore, the magnitude of which depends on the access, pore, surface charge, electroosmosis and potential leakage conductance. (c) Schematic illustration of the experimental devices. The silicon nitride film with 2 nm nanopores divides the liquid pool into aqueous *cis* and *trans* chambers. (d) Conductance and conductivity of HCl/NaCl at concentrations from 10^−7^ M to 1 M.

First, a 100 nm thick Si_3_N_4_ membrane was grown on a silicon wafer using low-pressure chemical vapor deposition. Then, a window on the other side of the wafer was opened using a wet etching process to expose the silicon nitride thin film. After the etching process, the silicon nitride film was exposed to a focused ion beam with high energy to reduce the film thickness to 20 nm. In the last step, a nanopore was drilled with a transmission electron microscope (TEM) using a tightly focused electron beam (Fig. 1a). To probe the electrodynamic properties of the ions, we must first investigate the composition and role of the nanopore conductance (Fig. 1b). Experimentally, the pore current is measured by inserting two electrodes into the aqueous *cis* and *trans* chambers (Fig. 1c). Using Ohm's law, the conductance can be obtained (Fig. 1d, see ESI†).

In order to investigate the concentration of nanopores (*R* ≫ *L*) in extremely thin films, we used molecular dynamics to simulate the passage of water and NaCl through graphene nanopores under an electric field. Based on the simulation results, we calculated the concentration distribution of ions along the radial and axial directions to compare with the bulk behavior. In order to investigate the effect of the electric field on the rearrangement of water molecules, we also calculated statistics for the electric potential distribution of pure water along the axis (see ESI†). The mutual confirmation of the experiment and simulation helped us to understand the transport function of ions inside the nanopore.

## 3. Results and discussion

### 3.1. Ensemble averaged concentration inside the nanopore

In a cylindrical pore, we can also simply express the conductance as 
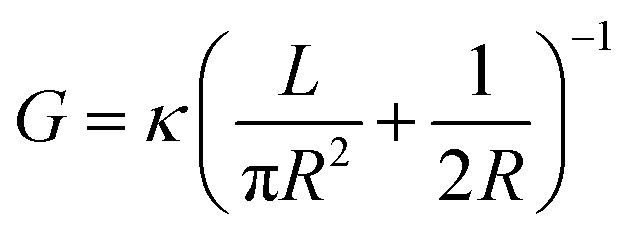
, where *G* and *κ* are the conductance and conductivity of an electrolyte inside the pore, respectively; *R* and *L* are the radius and the length of a nanopore, respectively. The conductivity can be written as 
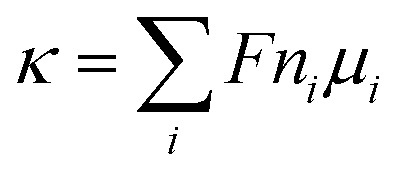
, where *n*_*i*_ and *u*_*i*_ are the concentration and the electrophoretic mobility of an ion of the *i*th species, respectively; *F* is the Faraday constant. Intuitively, the migration behavior of ions is determined by (6) the electrolyte concentration gradient and (7) ion mobility, but factors 6 and 7 are controlled by (5) the environmental pH and (8) electric field strength. Both theoretical studies^25–27^ using molecular dynamics and experimental nuclear magnetic resonance studies^16^ show that the ion concentration deviates from the bulk values. Various power-law relations between the conductance and ion concentration (*G* ∝ *n*_0_^*α*^) have been widely studied, which has also been verified in our experiments (Fig. 1d). Conductivity exhibits a similar phenomenon. Moreover, the conductivity of NaCl solution is always an order of magnitude lower than that of HCl solution, which is similar to the bulk behavior.

**Fig. 2:**
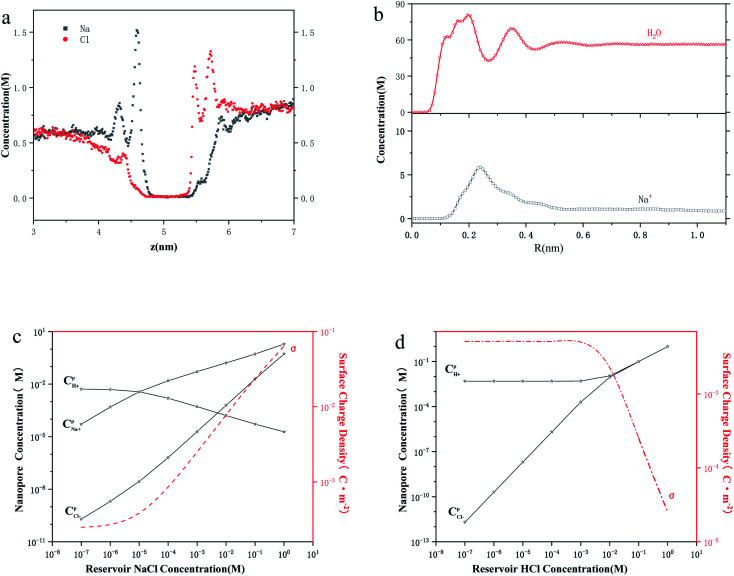
Ionic concentration inside the sub-nm nanopore. (a) and (b) Axial and radial distribution of the ion concentration from the molecular dynamics results. The system selects a cubic box as the research unit; the box size is *L*_*x*_ = 5.28 nm, *L*_*y*_ = 5.39 nm, *L*_*z*_ = 10.60 nm. Conditions: electric field strength *E* = 0.5 V nm^−1^, bulk concentration: 1 M, and nanopore diameter *D* = 2 nm, where *z* = 5.30 nm corresponds to the graphene sheet. (c) Ionic concentration inside the nanopore and surface charge density for NaCl solutions. Nanopore ionic concentration and surface charge density increase with increasing reservoir ion concentration. (d) Ionic concentration inside the nanopore and surface charge density for HCl solutions.

As shown in Fig. 2a, both sodium ions and chloride ions formed concentration polarization layers on either side of the thin-film nanopore, which is similar to the density profiles reported by Titus^27^ and Hu.^25^ In the polarization zone, the peak sodium ion and chloride ion concentrations are 1.55 and 1.33 times the bulk values. This difference may be caused by the difference in surface charge and ion hydration. The radial distribution of ions and water in Fig. 2b shows that a water and ion layer is formed inside the nanochannel, and two water layers surround the ion layer. The difference between the size-constraint effect^28,29^ of pure water passing through the nanochannel is that the peak concentration of water occurs close to the center of the hole, rather than the peak concentration of water being close to the wall in the nanochannel like in pure water. This behavior is obviously a consequence of the fact that the cations can optimize their hydration shell and quickly pass through the nanochannel. It is worth noting that the peak concentration of cations is five times the bulk value. These phenomena all imply that the ion concentration inside the nanochannel is different from the bulk concentration.

To further understand the role of concentration in the ion transport behavior, we use the model of ref. 12 to estimate the ion concentration inside the nanochannel. Here, we define the ensemble average concentration as the average concentration of all ions in the access conductance,^30^ surface conductance, potential leakage conductance,^31^ and nanopore conductance regions (Fig. 1b). n_OH^−^_/*n*_Cl^−^_/*n*_H^+^_/*n*_Na^+^_ are used to represent the concentrations of OH−/Cl−/H+/Na+, which are different from the bulk concentration *n*_0_. For a given surface charge density *σ*, the ion concentration inside the nanopore must satisfy a certain relationship due to the quasi-electroneutrality condition, as suggested by earlier works:^12^*σ*/*eR* = *n*_OH^−^_ + *n*_Cl^−^_ − *n*_H^+^_ − *n*_Na^+^_. As we performed the measurements under ambient conditions, the pH of the electrolyte solution was 5 due to CO_2_ absorption.^12,32^ By using the Donnan equilibrium condition to relate the electric potentials and Poisson–Boltzmann theory, we can obtain the ion concentration results (Fig. 2c and d). As shown in Fig. 1d and 2c, the ion concentration in the 2 nm nanopore changes drastically until the bulk concentration reaches 1 mM, although there is no change in the conductance value. The complete ion transport operation means that an ion can only be emitted when the ion is trapped in the channel. In this region, although the concentration of the pore increases rapidly, the surface charge also increases just as quickly, so that the ions are always in a state of depletion to maintain electrical neutrality and the conductance cannot be increased. In the interval from 1 mM to 100 mM, the proton concentration drops rapidly, resulting in an increase in surface charge,^12,33,34^ while the Na^+^/Cl^−^ concentration also increases rapidly with the increase of bulk concentration. Part of the Na^+^ participates in maintaining the electrical neutrality of the nanopore, and part of it is used for transportation. The increase in the concentration of chloride and sodium ions also leads to a power-law increase in conductance (*G* ∝ *n*_0_^*α*^).

To explore the role of the Na^+^ and H^+^ concentrations on the conductance, we performed the experiment using HCl alone to measure the transport through nanopore for comparison with the NaCl solution (Fig. 2d). This experiment shows that when the bulk concentration is higher than 1 mM, the surface charge drops rapidly, and the Cl^−^ and H^+^ concentrations are almost the same. A similar phenomenon has been reported, which is consistent with our conclusion that there is no surface charge below pH 2 due to the complete protonation of the silica surface.^35^ However, in Fig. 2c, it can be observed that Na^+^ concentration is at least one order of magnitude higher than that of Cl^−^ until the bulk concentration reaches 100 mM. Even if the bulk concentration reaches 1 M, the Na^+^ concentration (1.9 M) in the NaCl solution is almost four times the Cl^−^ concentration (0.53 M), while Cl^−^ and H^+^ concentrations (1 M) in the HCl solution are the same as the bulk concentration. Moreover, the Na^+^/H^+^ concentration in the NaCl/HCl solution is always higher than the bulk concentration. In contrast, the Cl^−^ concentration is always less than or equal to the bulk concentration. These phenomena show that the charged nanopores are ion-selective.

### 3.2. Ionic conductance

In many experiments that study ion dynamics in micron-sized channels, the ionic conductance measurement (for neutral pores) is estimated as^33,36^1
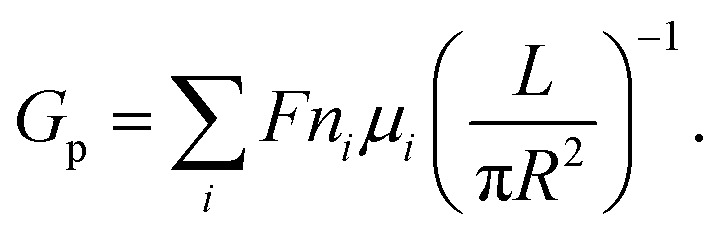
However, the conductance (*G*_p_) is only reasonably accurate for channels (*L* ≫ *R*) with a very high aspect ratio *L*/*R*, where the access resistance is negligible. When the ratio of film thickness to pore size is small (*L* ≈ *R*), the influence of entrance resistance on the system resistance cannot be ignored.^6,37–40^ The access conductance (*G*_a_) was derived theoretically by Hall^41^ and can be expressed as 
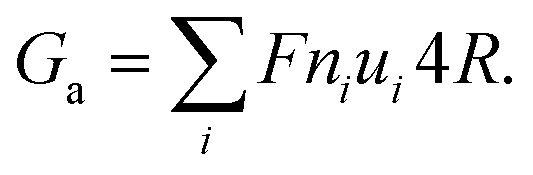
 The ionic conductance of ions can be theoretically predicted through a combination of access resistance and pore resistance as suggested by earlier works:^30,38,40,42^2
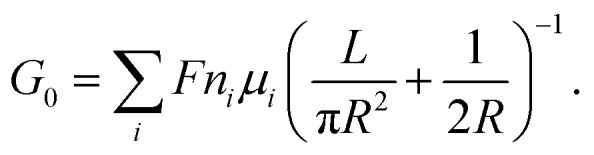


For a charged nanopore, the surface conductance makes an additional contribution to the overall conductance, as the nanopore requires counterions to screen the surface charge and maintain electrical neutrality.^3,43^ The surface conductance (*G*_s_) can be expressed as 
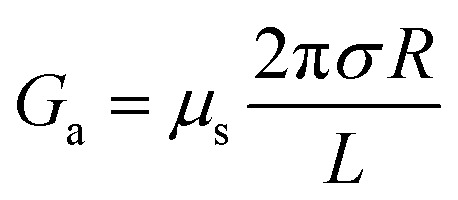
, where *μ*_s_ is the mobility of the counterions of a charged pore surface.^30^ The overall conductance (*G*_0_) can be written as *G*_0_^−1^ = *G*_a_^−1^ + (*G*_s_ + *G*_p_)^−1^. By combining eqn (1) and (2), the total conductance can be expressed as^31^3
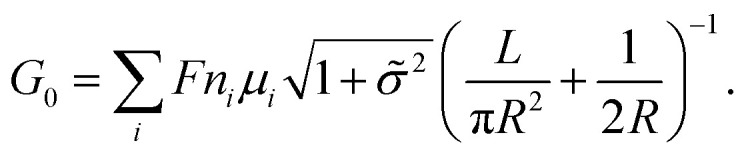
Here, the value of σ̃ is the ratio of the net charge concentration required in the pore and the charge concentration of the bulk solution, which is a dimensionless coefficient. For the experimental system in which a 1 : 1 solution is placed in a cylindrical pore with a homogeneous charge distribution, σ̃ can be written as 
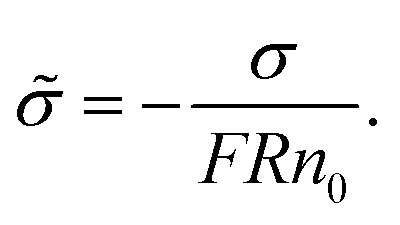


A large number of experiments have shown the failure of the principle of electrical neutrality inside the nanopore region, such as charge overspill, end effects, surface-electric-potential leakage and electroneutrality breakdown, but the entire nanopore system retains electroneutrality.^16,31,44–46^ The reason for these phenomena is that the surface charge potential leaks into the reservoir and the net charge inside the pores is insufficient. This potential leakage is different from the effect of surface charges, which enhances the counterion transport and weakens the co-ion transport. Taking the electric potential leakage conductance (*G*_l_) into consideration, the electrophoretic conductance (*G*_ph_) can be expressed as4
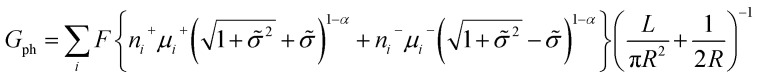
where *α* is the fraction of the surface-electric potential that leaks out of the pore.^31^ The total conductance of the nanopore should be composed of two parts: the electrophoresis conductance (*G*_ph_) and the electroosmotic (*G*_eo_) conductance affected water transport driven by the ionic migration. Then, the overall conductance (*G*_0_) can be written as^3,31^5
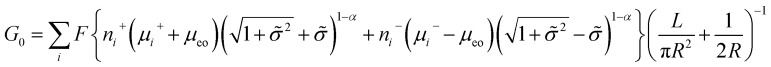


**Fig. 3:**
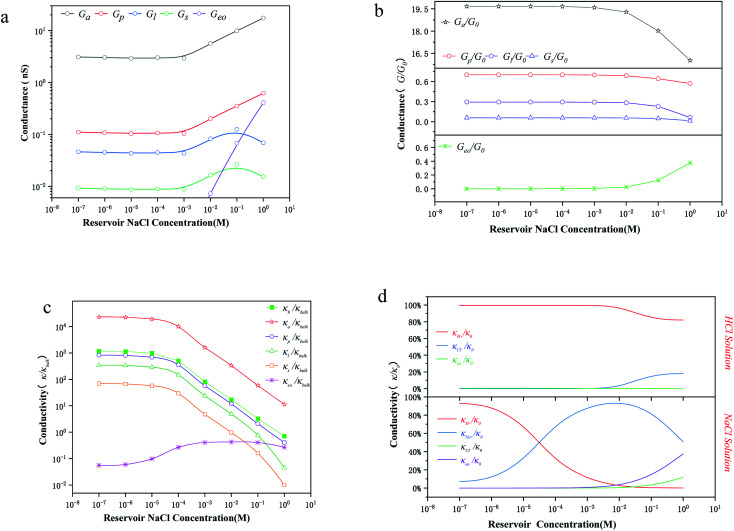
Ionic conductance, conductivity and ion selectivity for the sub-nm nanopore. (a) Comparison of the components of the total ionic conductivity in a 2 nm nanopore. The increase in bulk concentration also leads to a power-law increase in conductance (*G*_a_, *G*_p_, *G*_l_, *G*_s_ ∝ *n*_0_^*α*^), where *G*_a_, *G*_p_, *G*_l_, *G*_s_, and *G*_eo_ are the access, pore, surface charge, potential leakage and electroosmosis conductance, respectively. (b) Ratios of *G*_a_, *G*_p_, *G*_l_, *G*_s_, and *G*_eo_ to the total ionic conductivity (*G*_0_) as a function of reservoir concentration. (c) Ratio of conductivity in the nanopore (*κ*_a_, *κ*_p_, *κ*_l_, *κ*_s_, *κ*_eo_, *κ*_0_) to the corresponding bulk conductivity (*κ*_bulk_). Here, *κ*_a_, *κ*_p_, *κ*_l_, *κ*_s_, *κ*_eo_, and *κ*_0_ are the access, pore, surface charge, potential leakage, electroosmosis conductivity and the total ionic conductivity, respectively. (d) Ion selectivity for the sub-nm nanopore and comparison of the contributions of the different ions to the conductivity. *κ*_H^+^_, *κ*_Cl^−^_, and *κ*_Na^+^_ are the H^+^, Cl^−^, and Na^+^ conductivity, respectively

Fig. 3a shows the conductance values based on the experimental results (Fig. 1c). When the bulk concentration is less than 10 mM, the conductance values follow the order *G*_a_ > *G*_p_ > *G*_l_ > *G*_s_ > *G*_eo_, and an increase in the bulk concentration also leads to a power-law increase in conductance (*G*_a_, *G*_p_, *G*_l_, *G*_s_ ∝ *n*_0_^*α*^). Furthermore, the most conductive part is the access resistance, which is 1–2 orders of magnitude higher than the other parts. At the same time, it can be seen from Fig. 3b that the access conductance is 16–20 times the total conductance. This situation indicates that ions queue up for entrance into the nanopore and wait to pass through the nanopore, which results in a concentration polarization phenomenon, in accordance with the MD conclusion (Fig. 2a). For the nanopore itself, the pore conductance (*G*_p_), surface conductance (*G*_s_) and electric potential leakage conductance (*G*_l_) constitute the overall conductance of the nanopore (*G*_nc_), and the sum of these three partial values (*G*_p_, *G*_l_, *G*_s_) is close to the total conductance (*G*_0_) below 10 mM in Fig. 3b. This conclusion shows that *G*_nc_ is key to the conductive properties of the nanopore (*G*_0_). This is because in a series resistor, the larger the resistance value, the greater the voltage drop it bears. Moreover, the additional conductance (*G*_l_, *G*_s_) affected by the surface charge reaches half of the pore conductance (*G*_p_), which indicates that ionic transport through a charged nanopore at low ionic concentration is governed by the surface charge, consistent with earlier reports.^3,47–49^ At the same time, we also observed that the surface potential leakage conductance is about six times the surface conductance below 100 mM, which provides ideas for increasing the ion selectivity of the nanopore and accelerating ions in energy conversion devices. Slightly different from in Duan's^12^ report of a rectangular 2 nm nanochannel, the effect of the electroosmotic flow on the conductance starts at 10 mM instead of 100 mM in a cylindrical pore. This is because *G*_a_, *G*_l_, and *G*_s_ have an increased influence on the total conductance, which enhances ion transport and leads to an increase in electroosmotic flow. Research indicates that the two obvious characteristics of conductance inside the nanopore are the surface-charge-governed regimes at low ion concentrations and the bulk behavior regimes at high ion concentrations.^12,47^Fig. 3a illustrates that the additional conductance (*G*_l_, *G*_s_) affected by the surface charge reaches a peak value at 100 mM, which indicates that the leading role of the surface charge has begun to decrease, and that the role of electroosmotic flow has become stronger. In other words, 100 mM is the transition point between the surface-charge-governed and the bulk behavior regimes.

**Fig. 4:**
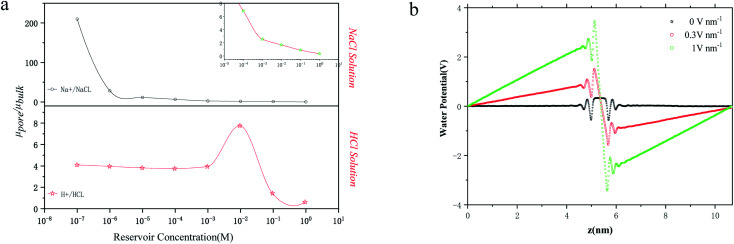
Ion mobility and water potential for the sub-nm nanopore. (a) Ion mobility and ratio of ionic mobility inside the nanopore (*μ*_pore_) to the corresponding bulk mobility (*μ*_bulk_) as a function of reservoir concentration. The inset in the figure is a partial enlarged view of the Na^+^ mobility, which shows that the change at low ionic concentration is still drastic. (b) Average axial electrostatic potential for H_2_O in a MD model with a 2 nm nanopore for electric field strengths of 0 V nm^−1^, 0.3 V nm^−1^, and 0.5 V nm^−1^, respectively. The stronger the electric field, the stronger the ability to rearrange water molecules.

To further investigate the transition point, we removed the geometric factors and studied the difference between the nanopore conductivity (*κ*_0_) and NaCl solution conductivity(*κ*_bulk_). Fig. 3c illustrates that 
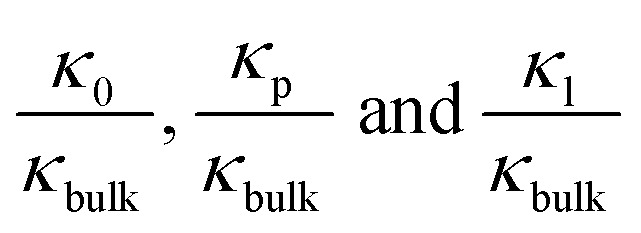
 decrease from 10^3^ to 10^0^ as the concentration is increased to 100 mM, which shows that the enhancement effect of surface charge on ion transport almost disappears after the transition point is exceeded. Moreover, these curves have a similar arrangement to the conductance distribution curve in Fig. 3a
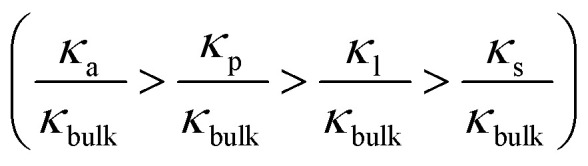
, and such a result is expected due to the relationship between conductance and conductivity. The access, pore and potential leakage conductivity (*κ*_a_, *κ*_p_, *κ*_l_) are 10^2^ to 10^4^ times the conductivity of the NaCl solution (*κ*_bulk_) below 10^−4^ M, while these values quickly drop to 10^0^ at concentrations of 10^−4^ M to 10^−1^ M. This different performance is caused by the different concentration and ion mobility behavior in the two regions (Fig. 2c and 4a): (1) at ∼10^−4^ M, the ionic concentration inside the pore increases rapidly due to the surface charge and the ion mobility inside the pore is much higher than the ion mobility in the NaCl solution; (2) between 10^−4^ M and 10^−1^ M, the increase in the ion concentration inside the pore is slower than the increase in the bulk concentration, and this increase in concentration also leads to a decrease in ion mobility.

Fig. 3d illustrates that the contribution of protons to the conductance exceeds 80% in HCl solution, even at a bulk concentration of 1 M. This is because the mechanism of proton transfer is tunneling, and its exceptionally large mobility produces a very high conductivity, which is different from metal ions.^5,12^ Therefore, in HCl solution, the electroosmotic flow contributes less than 20% to the conductivity, which can be ignored at low ion concentration. In NaCl solution, the conductivity in a charged nanopore at ∼10^−5^ M is governed by H^+^, and the transition to being dominated by Na^+^ occurs from 10^−4^ M to 10^−2^ M, but the conductivity is a collective effect of electroosmotic flow, H^+^ and Na^+^ when the ion concentration is higher than 10^−2^ M.

### 3.3. Ion mobility

Another important factor in ion transport is ion mobility. Using eqn (5), we compared the cation mobility in the sub-nm cylindrical nanopore with the bulk mobility (*μ*_bulk_) based on the experimental results. In HCl solution, the proton mobility of the nanopore 
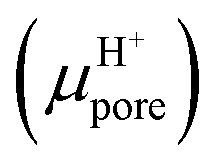
 was found to be four times the bulk proton mobility 
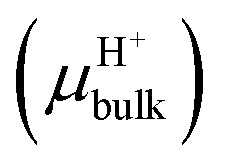
 until the bulk concentration reached 10^−3^ M; this conclusion is consistent with earlier reports^12^ (Fig. 4a). Moreover, we also found that the proton mobility reached a peak of seven times the bulk proton mobility at 10^−2^ M, and then quickly dropped below the bulk mobility value. The proton transport method first involves the formation of hydrate ions, and then tunneling for transport. Therefore, the ion mobility in this way is determined by the speed of rearrangement of water molecules.^5,50^ Obviously, an external electric field can strengthen the rearrangement of water, which is conducive to proton transmission. Our simulation results (Fig. 4b) also proved that the stronger the electric field, the more violent the orderly changes of the water potential, which reflects the relationship between the rearrangement of water molecules and the electric field. In the measured nanopore system, the voltage drop is mainly concentrated on the nanopore, which makes the electric field intensity in the nanopore particularly high (>10^7^ V m^−1^). This strengthening effect of the rearrangement increases the proton mobility, making it greater than the bulk mobility. As there is no surface charge below pH 2 due to the full protonation of the silica surface (Fig. 2d), the electrostatic interaction exerted by the electric double layer on the counterions disappears, so that the ion mobility reaches the peak value at 10^−2^ M (Fig. 4a). After the ion concentration exceeds 10^−2^ M, the Cl^−^ concentration (Fig. 2d) and its contribution to the conductance (Fig. 3d) increase to be comparable to those of the protons, and the transport of Cl^−^ has a destructive effect on the rearrangement of water molecules, causing the proton mobility to decrease.

Unlike the proton transport in HCl solution, the Na^+^ mobility in NaCl solution shows a downward trend, but it is always higher than the maximum value of bulk Na^+^ mobility (*μ*Na_^+^_ at infinite dilution is 5.19 × 10^−8^ m^2^ V^−1^ s^−1^) until a concentration of 10^−2^ M. The reason for this phenomenon is that the electric field strength inside the nanopore (2 × 10^7^ V m^−1^ in this experiment) is higher than the critical electric field of the Wien effect (10^7^ V m^−1^), which causes the Wien effect when ions pass through the nanopore.^50^

### 3.4. Ion selectivity

There is a significant difference in the ion exchange ability of the pore to pass/reject the two ion types, and this characteristic can be defined as the ion selectivity of the pore.^51,52^ Nanochannels/nanopores have shown the ability to replicate and mimic the characteristics for use in a range of applications, including ultra-sensitive ion detection and antimicrobial agents.^2,53^ The stronger the ion selectivity, the more ions will be used for transport through the nanopore, which leads to a greater contribution to the conductance. Therefore, the ion selectivity can be expressed as^31^6
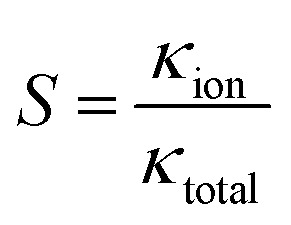


In HCl solution, the Na^+^ selectivity is always less than 50% at ∼10^−5^ M, and it reached a peak of 95.69% at 10^−2^ M as the bulk concentration was increased. In the concentration region below 10^−2^ M, it is the surface charge that plays the active role, which repels the ions of the co-ion (Cl^−^) and attracts the counterions (Na^+^) at the same time, thereby enhancing the Na^+^ selectivity. In the concentration region above 10^−2^ M, the Na^+^ selectivity decays, as the role of electroosmotic flow is beginning to manifest and its physical occupation hinders ion selectivity. Because surface charge and electroosmotic flow both reduce the selectivity toward chloride ions, the selectivity toward chloride ions is always at a low level.

## 4. Conclusions

In summary, we have investigated the ionic transport of a sub-nm nanopore from the perspective of conductance using MD and experimental methods. The results show that the ion transport behavior through the sub-nm nanopores (*L* ≈ *R*) on the film is different from that in the nanochannel (*L* ≫ *R*), and is even more different from the bulk behavior. Under the action of surface charge, the average ion concentration inside the nanopore is much higher than the bulk value. It is found that 100 mM is the transition-point between the surface-charge-governed and the bulk behavior regimes. Moreover, it is found that the conductive properties of the nanopore at low bulk concentration are determined by the surface charge potential leaks into the reservoir, based on investigation of the access, pores, surface charge, electroosmosis and potential leakage conductance. The results also exhibited up to a four-fold increase in proton mobility in HCl solution, and we concluded that this result is caused by the extremely high electric field strength accelerating the rearrangement rate of the water molecules. At the same time, in NaCl solution, there is a huge increase in Na^+^ mobility due to the Wien effect. The nanopores also showed a strong selectivity toward Na^+^ and a strong repellence for Cl^−^ due to the effect of surface charge and electroosmosis. These conclusions may help to provide a new understanding of the design of nanofluidic devices.

## Conflicts of interest

There are no conflicts to declare.

## Supplementary Material

RA-011-D1RA01089A-s001
