# Supplementary material for: Electric control of ionic transport in sub-nm nanopores
Source: RSC Adv. 2021 Apr 13;11(23):13806–13. doi: 10.1039/d1ra01089a (PMC8697696; doi:10.1039/d1ra01089a)
Supplement: RA-011-D1RA01089A-s001 [file RA-011-D1RA01089A-s001.pdf]

Electronic Supplementary Material (ESI) for RSC Advances.
This journal is © The Royal Society of Chemistry 2021

Supporting Information

Electric control of Ionic transport in Sub-nm Nanopore

Anping Ji, [a, b] Yunfei Chen [*a]

a. School of Mechanical Engineering and Jiangsu Key Laboratory for Design and Manufacture of Micro-Nano Biomedical Instruments, Southeast University, Nanjing, 211189.

E-mail: ji-anping@sanxiau.edu.cn

b. School of Mechanical Engineering, Chongqing three gorges university, Chongqing, 404100

1. Experiment detail

Fabrication of sub-nm nanopore. The semiconductor material silicon nitride, which is one of the most widely used materials in the manufacture of nanofluidic devices with mature manufacturing technology, is selected as the research object. First, a 100 nm thick Si_3N_4 membrane was grown on the silicon wafer by low pressure chemical vapor deposition. Then, a window on the other side of the wafer was opened using wet etching process to expose the silicon nitride thin film. After the etching process, the silicon nitride film was exposed to a focused ion beam with high energy to reduce the film thickness to 20 nm. At the last step, a nanopore can could be drilled by the transmission electron microscope (TEM) beam operated at 300kV. The fabricated nanopore size could be tuned using the electron beam with low intensity.

Preparation before measuring. First, it is necessary to clean the wafer on which the nanopore is fabricated with piranha solution to remove any contaminants. The silicon nitride film with 2-nm nanopores divides the liquid pool into aqueous cis. and trans. chambers. Ag/AgCl electrodes immersed on both sides of the pore are connected to a patch clamp amplifier (HEKA EPC 10 USB, HEKA Instruments) to measure the ionic current with picoampere sensitivity. The device was placed in a double faraday cage to reduce electrical noise.

Ion current measurement in sub-nm nanopore. We add the degassed and filtered salt solution to the liquid pool, and then wait until it reaches a stable state before starting the experiment. By sweeping the voltage from -500mV to 500mV at a scan rate of 100 mV per 2 seconds, we can measure many sets of current data. During the measurement, we increase the bulk concentration from low to high (10^{-7} M ~ 10^{0} M). By measuring the different concentrations of the ion current-voltage curve, the fitted value of the ion conductance in the channel can be obtained. According to the conductance value and the continuous theoretical model, the conductance composition and ion mobility can be obtained by calculation.

2. Molecular dynamics

In this study, ion concentration polarization layer is investigated with molecular dynamic (MD) simulation. A highly efficient MD package GROMACS was performed to study the influence of electrical field, ion concentration and diameter of nanopore.

Simulation model for 2-nm nanopore. The system selects a cube box as the research unit, the box size is: L_x*=5.28 nm,* L_y*=5.39 nm,* L_z*=10.60 nm* .The conditions is electric field strength E=0V nm^{-1}, 0.3V

nm^{-1}, 0.5V nm^{-1}, 1V nm^{-1}, the NaCl concentration 0.1M,0.5M,1M, and nanopore diameter D=2nm, where *z=5.30nm* corresponds to the graphene sheet. The bond length between carbon atoms is 0.142 nm, and the box is filled with TIP3P model water molecules. The system model is completed by the cooperation of the software GROMACS 5.02 and VMD(Figure S1).

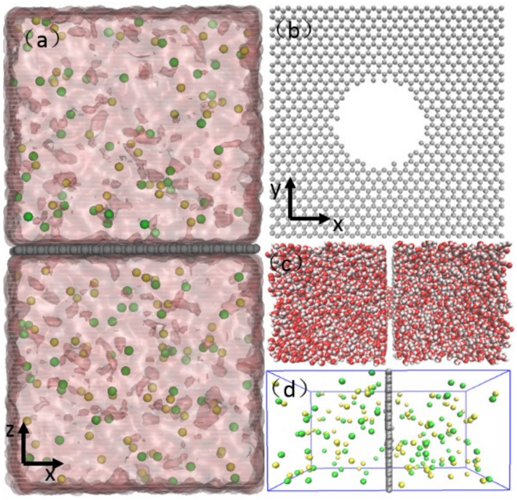

Figure S1.Water and ions passing through graphene nanopore. (a).Schematic diagram of water and ions passing through graphene nanopore model.(b).graphene nanopore; (c). Water molecule model. (d). Ions and graphene.

Simulation parameter setting. All parameter settings refer to earlier reports[1,2]. After the experiment is over, a comparison with some of the reported results verifies the feasibility of the experiment. The system temperature is stable at 300 K. The pressure is at 1 atmosphere. The temperature coupling method is the V-rescale method, and the pressure coupling method is the Parrinello-Rahma method. The simulation uses the OPLS all-atom force field, and the van der Waals interaction between ions is calculated by the Lennard-Jones (LJ) model. The cutoff radius is 1.0nm, while the Coulomb electrostatic interaction is calculated by the PME (particle-mesh Ewald) method. The cutoff distance is 1.0 nm. In order to drive the ions to move directionally in the system, a uniform electric field is applied in the Z direction. The atoms along the four edges of graphene are fixed. The time interval of each step of the system simulation is 2 fs, and the total number of steps is 5 million steps. The data storage frequency is 0.2 ps, and the total running time of the system is 10 ns.

3. Theoretical model for the ensemble averaged concentration inside the nanopore

For a given surface charge density σ, the ion concentration inside the nanopore must satisfy a certain relationship due to the quasi-electroneutrality condition, as suggested by earlier works[3]:

$$\frac{\sigma}{eR} = n_{OH^-} + n_{Cl^-} - n_{H^+} - n_{Na^+} . \tag{1}$$

$n_{OH^-}/n_{Cl^-}/n_{H^+}/n_{Na^+}$ are used to represent the concentration of $OH^-/Cl^-/H^+/Na^+$, which are different from bulk concentration n_0. By using the Donnan equilibrium condition to relate electric potentials and Poisson-Boltzmann theory, we can get such a relationship:

$$\frac{n_{ion}}{n_{bulk}} = exp\left\langle \frac{-e}{kT}(\phi_{ion} - \phi_{bulk}) \right\rangle. \qquad (2)$$

For silicon nitride film, the surface charge is related to the PH value, and its value can be expressed as

$$\sigma = \frac{\sigma_0 K_d}{K_d + n_{H^+}} \qquad (3)$$

σ_0 is the maximum possible charge density(-1.28$C \cdot m^{-2}$ in this experiment), and Kd is the equilibrium dissociation constant(equal to 10^{-6} M[10])[3,4].

4. Electroosmotic and ion mobility

Electroosmotic mobility. The electroosmotic mobility derived by Biesheuvel and Bazantusing SC theory and the Donnan equation can be expressed as[5,6]

$$\mu_{eo} = \frac{-\sigma R}{4\eta} \qquad (4)$$

where η is the viscosity of the solution. η can be obtained from the Stokes equation.

Ion mobility. In this paper, we assume that the transport of different ions in the nanopore has a similar relationship with the transport of bulk behavior. For fully dissolved sodium chloride solution, ionic mobility in a bulk solution can be obtained, as suggested by earlier works:

$$\mu_{bulk} = \frac{\mu_{bulk}}{1 + \frac{0.508 * \sqrt{I_z}}{3.29 * \alpha\sqrt{I_z}}} \qquad (4)$$

The above equation applies to ambient temperature at 25℃, $I_z = \frac{1}{2}\sum_j n_j^2$ is the ionic strength, and α is an adjustable parameter related to the ion size (Cl-, a ≈ 0.3 nm; Na+, a ≈ 0.4 nm)[3].

References

1 Suk *et al.* Ion transport in sub-5-nm graphene nanopores. *Journal of Chemical Physics* **140**, 084707-084701-084707-084707 (2014).

2 Hu, G., Mao, M. & Ghosal, S. Ion transport through a graphene nanopore. *Nanotechnology* **23**, doi:10.1088/0957-4484/23/39/395501 (2012).

3 Duan, C. & Majumdar, A. Anomalous ion transport in 2-nm hydrophilic nanochannels. *Nat. Nanotechnol.* **5**, 848 (2010).

4 Van, d. H., Frank H. J., Stein, D. & Dekker, C. Streaming currents in a single nanofluidic channel. *Physical Review Letters* **95**, 116104 (2005).

5 Biesheuvel, P. M. & Bazant, M. Z. Analysis of ionic conductance of carbon nanotubes. *PHYSICAL REVIEW E* **94**, 050601 (2016).

6 Noh, Y. & Aluru, N. R. R. Ion Transport in Electrically Imperfect Nanopores. *ACS Nano* **XXXX**

(2020).
